# Effectiveness and cost-effectiveness of a blended exercise intervention for patients with hip and/or knee osteoarthritis: study protocol of a randomized controlled trial

**DOI:** 10.1186/1471-2474-15-269

**Published:** 2014-08-08

**Authors:** Corelien JJ Kloek, Daniël Bossen, Cindy Veenhof, Johanna M van Dongen, Joost Dekker, Dinny H de Bakker

**Affiliations:** Tilburg University, Tranzo, PO Box 90153, Tilburg, LE, 5000 The Netherlands; Netherlands Institute for Health Services Research (NIVEL), Utrecht, The Netherlands; Department of Health Sciences, EMGO Institute, VU University Medical Center Amsterdam, Amsterdam, The Netherlands; Department of Rehabilitation Medicine & Department of Psychiatry, EMGO Institute, VU University Medical Center Amsterdam, Amsterdam, The Netherlands

**Keywords:** Osteoarthritis, Physical activity, Blended care, e-Health

## Abstract

**Background:**

Exercise therapy in patients with hip and/or knee osteoarthritis is effective in reducing pain, increasing physical activity and physical functioning, but costly and a burden for the health care budget. A web-based intervention is cheap in comparison to face-to-face exercise therapy and has the advantage of supporting in home exercises because of the 24/7 accessibility. However, the lack of face-to-face contact with a professional is a disadvantage of web-based interventions and is probably one of the reasons for low adherence rates. In order to combine the best of two worlds, we have developed the intervention e-Exercise. In this blended intervention face-to-face contacts with a physical therapist are partially replaced by a web-based exercise intervention. The aim of this study is to investigate the short- (3 months) and long-term (12 months) (cost)-effectiveness of e-Exercise compared to usual care physical therapy. Our hypothesis is that e-Exercise is more effective and cost-effective in increasing physical functioning and physical activity compared to usual care.

**Methods/Design:**

This paper presents the protocol of a prospective, single-blinded, multicenter cluster randomized controlled trial. In total, 200 patients with OA of the hip and/or knee will be randomly allocated into either e-Exercise or usual care (physical therapy). E-Exercise is a 12-week intervention, consisting of maximum five face-to-face physical therapy contacts supplemented with a web-based program. The web-based program contains assignments to gradually increase patients’ physical activity, strength and stability exercises and information about OA related topics. Primary outcomes are physical activity and physical functioning. Secondary outcomes are health related quality of life, self-perceived effect, pain, tiredness and self-efficacy. All measurements will be performed at baseline, 3 and 12 months after inclusion. Retrospective cost questionnaires will be sent at 3, 6, 9 and 12 months and used for the cost-effectiveness and cost-utility analysis.

**Discussion:**

This study is the first randomized controlled trial in the (cost)-effectiveness of a blended exercise intervention for patients with osteoarthritis of the hip and/or knee. The findings will help to improve the treatment of patients with osteoarthritis.

**Trial registration:**

NTR4224.

## Background

Osteoarthritis (OA) is worldwide one of the leading causes of pain and disability. Most common affected sites are the hip and knee joints [1]. In the United States, prevalence of knee OA for patients of 45 years or older is 17 percent and prevalence of hip OA 10 percent [2]. In the Netherlands, it is estimated that 312.000 persons suffer from knee OA (19.1/1000) and 238.000 from hip OA (14.5/1000) [3]. Presumably, these prevalence rates are underestimated since these data are solely based on general practice patients’ registrations [3]. OA is an age-related disease and besides pain and disability characterized by morning stiffness, reduced range of motion, instability of the joint and loss of health related quality of life [4, 5]. These symptoms induce that people with hip and/or knee OA are physically less active than the general population [6, 7]. In the long term, physical inactivity may lead to functional decline and psychological problems [8, 9].

Exercise therapy is the widely recommended non-pharmacological intervention in patients with hip and/or knee OA [10–13]. Therapeutic exercise, most of the time provided by a physical therapist, can consist of strengthening exercises, functional task-oriented exercises and/or aerobic training [10]. Many studies have shown the effectiveness of exercise therapy on patients’ physical functioning in daily life, for example stair climbing, rising from a chair or getting in or out a car [14, 15]. Besides, exercise therapy is effective in reducing patients’ levels of pain and increasing their physical activity [14, 15]. Unfortunately, the face-to-face contacts with a physical therapist are costly and a burden for the health care budget. To illustrate, Dutch healthcare costs related to OA were about 1,112 million euro in 2011 [16]. Likewise, the prevalence of hip and knee OA is expected to increase with 52% in 2040, due to the aging population and an increasing number of obese people [3]. In order to regulate OA costs there is a need for more (cost)-effective strategies to manage hip and/or knee OA.

The internet has created new possibilities to combine face-to-face care with online care, called blended healthcare [17]. The partial substitution of a web-based intervention for exercise therapy sessions is hypothesized to result in a (cost)-effective intervention in many ways. In the first place, a blended intervention will result in lower costs since the average number of physical therapy sessions for patients with OA will decrease. A second advantage of a blended intervention is the 24/7 online support for exercises at home. Support in exercises at home is important since adherence to self-directed exercise is a common problem in exercise therapy [18, 19]. Research highlighted the importance of adherence to exercises at home, since this positively influences treatment effects on pain and physical functioning [19]. Third, a well-designed web-based intervention in which patients’ can report their experiences with home exercises provides physical therapists information about patients’ individual needs for guidance.

Up till now, previous research in web-based interventions has focused on interventions without human support. Unfortunately, the effects of these interventions are small, especially in the long-term [20–24]. These modest effects can partly be explained by the absence of personal guidance [17]. To illustrate, in the study by Bossen et al. [23], patients cited that the lack of face-to-face contact in a self-guided web-based intervention was an important reason to discontinue. Hence, the combination of a web-based intervention with face-to-face contact has been recommended by several researchers [20, 25, 26]. To date, there are no studies investigating the (cost)-effectiveness of a blended intervention in the physical therapy setting. Therefore, we have developed e-Exercise and have planned to evaluate and implement this blended intervention. The intervention will be based on the Dutch “KNGF guideline OA hip-knee” for physical therapists [10]. The web-based part will be an adapted version of the previously developed and evaluated online PA program Join2Move [24], a web-based intervention for OA patients without support of a physical therapist. The aim of this study is to determine the (cost)-effectiveness of e-Exercise compared to usual care (physical therapy). Our first hypothesis is that e-Exercise will be more effective in terms of increasing PA and improving physical functioning in patients with hip and/or knee OA as compared to usual care. The second hypothesis is that e-Exercise will be cost-effective in comparison to usual care by physical therapists. The research question of this RCT study is: What is the short- (3 months) and long-term (12 months) (cost)-effectiveness of e-Exercise in patients with hip and/or knee OA on PA and physical functioning in comparison to usual care?

## Methods/Design

### Study design

**Figure 1:**
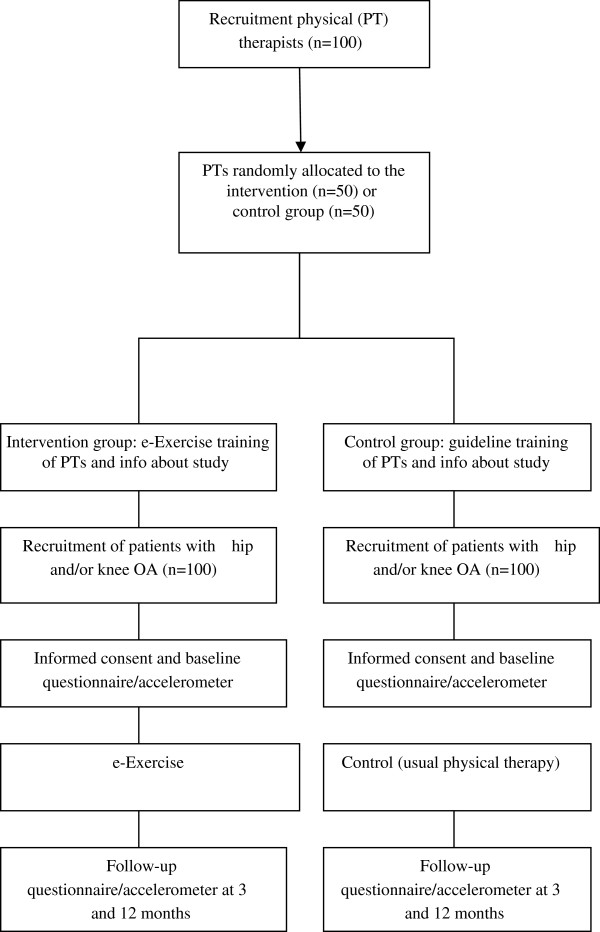
**RCT study procedures.**

A prospective, single-blinded, multicenter cluster randomized controlled trial (RCT) will be conducted. The study has been approved by the Medical Ethical Committee of the St. Elisabeth hospital Tilburg, the Netherlands (Dutch Trial Register NTR4224). The e-Exercise intervention will be compared with usual care (i.e. physical therapy). A flow diagram of the study protocol is shown in Figure 1.

### Participants

#### Physical therapists

A stratified random sample of 800 physical therapy practices in three provinces of the Netherlands (e.g. Noord-Holland, Utrecht and Gelderland) will be invited by letter to participate in the study. Contact information of physical therapy practices will be obtained from the national database for physical therapists of the Netherlands Institute for Health Services Research (NIVEL). Additionally, a recruitment advertisement will be placed in the online newsletter of The Royal Dutch Society for Physical Therapy (KNGF). Each participating physical therapy practice will be asked to enroll one or two physical therapists. The researchers will recruit 100 physical therapists. Inclusion criteria for physical therapists will concern: (i) practicing in primary care, (ii) treating at least six patients with OA of the hip and/or knee each year. Physical therapists practicing in another physical therapy practice participating in the study will be excluded.

#### Patients

In order to include 200 participants, each physical therapist is requested to recruit about two patients. Since the study of Veenhof et al. [27] showed that recruitment of OA patients in the physical therapy practice is difficult and research has shown that different recruitment strategies do not affect treatment outcomes [28], this study uses various recruitment strategies. First, patients with hip and/or knee OA who visit a physical therapy practice will be invited to participate in the study. Second, recruitment advertisements will be placed in local newspapers. Third, information letters and flyers will be sent to general practitioners. Responders to these articles and flyers will be allocated to the nearest participating physical therapist. Eligibility criteria of patients interested in the study concern: (i) age 40–80 years, (ii) OA of the hip and/or knee according to the clinical criteria of the American College of Rheumatology (ACR) [29]. Exclusion criteria will include: (i) being on a waiting list for a hip or knee replacement surgery, (ii) being contra-indicated for PA without supervision, (iii) being sufficiently physically active according to the physical therapist, (iv) participation in a physical therapy and/or PA program in the last six months, (v) no access to internet, (vi) inability to understand the Dutch language. The diagnostic ACR clinical criteria for knee OA are: knee pain and at least three of the following six criteria: age > 50 years, morning stiffness <30 minutes, crepitation, bony tenderness, bony enlargement and no palpable warmth. Diagnostic ACR clinical criteria for hip OA are: hip pain and hip internal rotation < 15 degree and hip flexion ≤ 115 degree; or hip internal rotation ≥ 15 degree and pain on hip internal rotation and morning stiffness of the hip ≤ 60 minutes and age > 50 years [29]. Contra-indications for PA will be determined using the adapted Physical Activity Readiness Questionnaire (PAR-Q) [30]. This questionnaire is used to identify patients for whom PA is inappropriate.

### Study procedure

Physical therapists willing to participate in the study will be screened on in- and exclusion criteria by a researcher (CK). Cluster randomization will be performed at the level of the participating physical therapy practices that will randomly be assigned to the intervention (e-Exercise) or the control group (usual care) by means of a computer-generated random sequence table. Physical therapists will receive a half day training about e-Exercise and the study procedure (intervention group) or about practicing according to the “KNGF guideline OA hip-knee” [10] and the study procedure (control group). Physical therapists will inform eligible patients about the study and screen them on in- and exclusion criteria. All suitable patients will be stimulated to contact the research team by telephone, e-mail or reply card. After an informative phone call with one of the researchers (CK or DB), interested patients will receive a letter about the trial and a request to complete an informed consent form. Patients recruited by the additional recruitment strategies (i.e. advertisements in newspapers and flyers from the general practitioner) will be informed by the researchers before they visit their physical therapist. Physical therapists in both groups will be asked to record the content of their treatment on a registration form. During the study period, both patient groups will continue their medication and usual care managed by other caregivers.

### Blinding

In this single-blind study , the physical therapists are not blinded since they will treat patients according to the randomization. The researchers will be blinded to group allocation until completion of the statistical analyses. Participants will be assigned to a unique digital trial code to ensure that treatment outcome measurement and statistical analysis will be performed blind to treatment allocation. Patient information will be stored in a separate database.

### Interventions

#### e-Exercise

The 3-month program e-Exercise is based on the Dutch guideline for physical therapists (10) and is a combination of (i) maximum five face-to-face sessions with a physical therapist, and (ii) a web-based PA intervention. Table 1 provides an overview of the program content of e-Exercise.

**Table 1:** **Description e-Exercise intervention**

Intake	Physical therapist	Anamnesis and physical examination
		Assessment in- and exclusion criteria
		Providing information about osteoarthritis, e-Exercise and study
		Scheduling a follow-up appointment for week 1
	Patient	Reading patient information letter
		Signing an informed consent
		Completing baseline measurement
Start e-Exercise		
Week 1	Physical therapist	Providing information about osteoarthritis and e-Exercise
		Providing information about the 3-day baseline self-test
		Instruction of the 4 stability/mobility exercises
	Physical therapist & Patient	Registration on website to participate in e-Exercise
		Online selection of central activity and 4 stability/mobility exercises
		Providing information about the 3-day baseline self-test
		Scheduling a follow-up appointment for week 2
	Patient	Signing online treatment agreement
		Performance of a 3-day baseline test
		Performance of 4 stability/mobility exercises
Week 2	Physical therapist	Providing information about physical activity and pain
	Physical therapist & Patient	Evaluation results from the 3-day self-test
		Determining short-term goal
		Discussing the gradual increase of the selected activity
		Evaluation stability/mobility exercises
		Scheduling a follow-up appointment for week 6
	Patient	Performance of online module 1, each module consists of:
		- Gradually increase selected activity
		- Video home exercises
		- Video/text self-management themes
Week 3-5	Patient	Online modules 2-4
Week 6	Physical therapist & Patient	Evaluation online modules 1-4
		Discussing the upcoming steps and weeks
		Evaluation stability/mobility exercises
		If necessary, scheduling an additional treatment between week 7-11
		Scheduling a follow-up appointment for week 12
	Patient	Online module 5
Week 7-11	Patient	Online modules 6-10
Week 12	Physical therapist	Discussing long-term goals
		Support to maintain a physically active lifestyle
	Patient	Online module 11

#### Face-to-face sessions

During the first face-to-face session (week 1), physical therapists will provide information about OA, the importance of PA and the relation of PA with pain. Together with their physical therapist, patients choose one physical activity, for example, walking, cycling or swimming. Physical therapists select and instruct four strength & stability exercises. Patients are instructed to perform the first module of the web-based part of the intervention. In this module, the patients will be asked to determine their physical load ability based on a 3-day self-test. The second assignment is the execution of strength & stability exercises. During the second face-to-face session (week 2), patients’ physical load ability will be discussed and personal short and long-term goals will be formulated according to the principles of Goal Setting, which is based on the idea that goals can affect action [31]. The strength & stability exercises will be trained again. After the second appointment, patients are instructed to perform four online modules for the duration of four weeks. In week 6, a third face-to-face treatment takes place. Patients’ progress will be discussed, based on an online report which is automatically sent to the physical therapists. This report contains a summary of website-visits and patients’ experiences with the strength and stability exercises. After the third face-to-face treatment, patients perform another six online modules. The final face-to-face appointment will take place in week 12. In this final treatment physical therapists will support and encourage patients to maintain a physically active lifestyle. If necessary, physical therapists can plan an additional fifth session. This optional session is especially for patients who are less capable to perform unsupervised physical exercises. Physical therapists are recommended to treat patients according to the e-Exercise protocol, however, with respect to their clinical competences, physical therapist are free to deviate from the protocol.

#### Web-based PA intervention

The web-based part of e-Exercise is based on the web-based intervention Join2move [32] and consists of three topics: (i) Graded Activity; the duration of patients’ chosen physical activity (e.g. walking, cycling, swimming) will gradually be increased until patients reach their personal short-term goal. (ii) Strength & Stability; each module contains two exercises. The number of repeats will gradually increase per 4 weeks. (iii) Information; topics about OA, PA, aetiology of OA, pain-management, weight-management, motivation, medication and social influences on pain will be discussed. Automatic emails are generated if participants do not visit the website once a week.

#### Usual care

Patients in the control group will receive usual care. For the current study, usual care is defined as any treatment provided by physical therapists. Physical therapists will be encouraged to practice according to the “KNGF guideline OA Hip-Knee” [10]. According to the guideline, the physical therapy treatment comprises the same three elements as e-Exercise: (i) information, (ii) physical exercise and (iii) strength and stability exercises. Practical content considerations can be made by therapists themselves. The number of sessions will differ per patient. From the NIVEL Primary Care Database we know that the average number of physical therapy sessions in patients with OA is 17.1 [33].

### Measurements

Three online questionnaires (0, 3 and 12 months) will be used for data collection. Participants will receive an accelerometer for the measurement of objective PA (0, 3 and 12 months). The physical therapists will measure physical functioning objectively at baseline and post-treatment (3 months). In addition, online cost questionnaires will be sent (0, 3, 6, 9 and 12 months). We offer no financial incentives to complete questionnaires or to wear accelerometers. Table 2 gives a summary of all measures that will be collected.

**Table 2:** **Summary of measures to be collected**

Primary outcome measures	Data collection instrument	Collection points
Physical functioning	HOOS and/or KOOS	0, 3, 12 months
	Timed “Up & Go” test	0, 3 months,
Physical activity	SQUASH	0, 3, 12 months
	ActiGraph GT3X tri-axial accelerometers	0, 3, 12 months
**Secondary outcome measures**		
OA related costs	Cost questionnaire	3, 6, 9, 12 months
Health related quality of life	EQ-5D	0, 3, 12 months
Self-perceived effect	7-point Likert scale	3, 12 months
Pain	NRS	0, 3, 12 months
Tiredness	NRS	0, 3, 12 months
Self-efficacy	Arthritis self-efficacy Scale	0, 3, 12 months
**Other measures**		
Age	Questionnaire	0 months
Sex	Questionnaire	0 months
Height	Questionnaire	0 months
Weight	Questionnaire	0, 3, 12 months
Educational level	Questionnaire	0 months
Location of OA	Questionnaire	0 months
Disease duration	Questionnaire	0 months
Presence of comorbidities	Questionnaire	0 months
Adherence	Completed web-modules	During intervention

#### Primary outcome measures

*Physical functioning* will be assessed subjectively with the subscale ‘function in daily living’ of the Hip OA Outcome Score (HOOS) [34] and/or the Knee Injury and OA Outcome Score (KOOS) [35], depending their affected joint. The HOOS and the KOOS assess 5 indicators: pain, symptoms, physical function, sport and recreation function and quality of life, in relation to patients’ hip or knee complaints. Each indicator is scored on a 5-point Likert scale (0 = extreme symptoms/problems; 4 = no symptoms/problems). A lower score indicates respectively more pain, symptoms, problems in physical functions, problems in sports and recreation activities and a lower quality of life. In addition, objectively physical functioning will be measured by the physical therapist with timed “Up & Go” test (TUG) [36]. In this easily administered test, the patient is requested to rise from an arm chair, walk three meters, turn, walk back again and sit down. Meanwhile, the physical therapist observes the patient and measures the time.

*Physical activity* will be measured subjectively with the SQUASH [37]. The questionnaire measures habitual PA during a normal week over the last few months. The total score is expressed as minutes per week. In addition, data can also be analysed according to whether the activity is light, moderate or vigorous. Objective PA will be measured through ActiGraph GT3X tri-axial accelerometers. Participants will be instructed to wear the monitor on a belt around their waist for five executive days [38], except during sleeping, showering or swimming. In addition, participants will be requested to fill out a short activity diary. This diary contains questions about wearing time, unusual activities and reasons for device removal. When accelerometers and diaries are returned by post, data can be downloaded, processed and subsequently analyzed. The widely accepted PA thresholds of Freedson et al. [39] will be used: 0–99 counts for sedentary activities, 100–1951 for light PA, 1952–5724 moderate PA, 5725–9498 for vigorous PA and 9499- max for very vigorous activities. The total time spent in light, moderate and vigorous PA will be summed and subsequently divided by the number of days worn to compute the daily average time spent in total activity. For analysis, data will be recorded at 1-minute intervals.

#### Secondary outcome measures

*Information on the patients’ healthcare utilization, (unpaid) productivity losses, and sports costs due to OA* will be gathered with four retrospective 3-month cost questionnaires that cover the full 12-months of the program. Healthcare utilization due to OA comprises of visits to a physical therapist, general practitioner, massage therapist, alternative therapist, medical specialist, as well as informal care, hospital care, the use of both prescribed and over the counter drugs and medical devices. Healthcare utilization will be valued using Dutch standards costs [40]. If these are unavailable, prices reported by professional organizations will be used. Medication use will be valued using unit prices derived from the “Royal Dutch Society of Pharmacy” [41]. Unpaid productivity losses will be valued in accordance with the “Dutch Manual of Costing” [40]. Paid productivity losses comprise of both sickness absence and presenteeism (i.e. reduced productivity while at work). Sickness absence will be valued in accordance with the “Friction Cost Approach” (FCA), with a friction period of 23 weeks and an elasticity of 0.8, using age- and gender-specific price weights [40]. The FCA assumes that production losses are confined to the “friction period” (i.e. time needed to replace a sick worker) and that a 100 percent loss of labour input corresponds with an 80 percent reduction in productivity (i.e. an elasticity of 0.8) [42]. The participants’ level of presenteeism will be measured using the “World Health Organization – Work Performance Questionnaire” as well as the “Productivity and Disease Questionnaire”, and valued using age- and gender-specific price weights [40, 43–45]. The cost of the e-Exercise intervention will be estimated using a bottom-up micro-costing approach [46].

*Health Related Quality of Life* will be measured with the EuroQol-5D (EQ-5D) [47]. This questionnaire comprises of 5 dimensions i.e., mobility, self-care, usual activities, pain/discomfort and anxiety/depression. Per dimension, patients are asked to indicate their health state on a 3-point Likert scale (1 = no problems; 3 = extreme problems). The questionnaire differentiates between 245 health states. These health states will be converted into utility units by using the Dutch tariff [48]. Utilities represent quality of life into a single number that ranges from 0 (death) to 1(full health). Quality adjusted life years (QALY’s) will subsequently be calculated by multiplying the participants’ health state utilities by the duration of time they spent in that particular health state.

*Self-perceived effect* will be assessed by a single question about the degree of change in osteoarthritis symptoms since their previous assessment. Patients will score this effect on a 7-point Likert scale (1 = much worse; 7 = much better). A higher score indicates a better self-perceived effect.

*Pain and tiredness* will be measured with a numeric rating scale (NRS; 0 is no pain/not tired and 10 is worst possible pain/very tired). Furthermore, pain will be assessed with the pain subscale of the HOOS and/or the KOOS [34, 35].

*Self-efficacy* will be measured by the Arthritis Self-efficacy Scale (ASES) [49]. Subscales for the ASES are pain, symptoms and physical functioning, the 19 statements can be scored on a 5 point-Likert scale (1 = fully disagree; 5 = fully agree). A higher score indicates more self-efficacy.

#### Other measures

*Adherence* will be measured objectively by quantitative data about usage which is automatically stored on the backend of the website. Usage is defined as completed week modules. Subjective adherence is measured by a questionnaire about patients’ adherence to the Graded Activity modules and Strength & Stability exercises (frequency and intensity).

*Content of physical therapy sessions* will be measured trough registrations forms, developed by the researchers. The registrations forms collect information about the adherence and content of the sessions.

*Patient characteristics* i.e. age, sex, height, weight, educational level, location of OA, disease duration and the presence of comorbidities will be assessed at baseline.

### Sample size

The power calculation is based on a previous multicenter cluster RCT study among patients with hip and/or knee OA [27] and performed for the primary outcome measure physical functioning (power 0.8; alpha 0.05). In this current RCT study, the target sample size will be 200 participants to detect a small to medium effect size (0.2-0.4) in physical functioning at a 2-sided significance level of 0.05 and anticipating on maximum loss to follow up of 20%.

### Statistical analysis

Descriptive statistics will be used to describe the main characteristics of the study population and to explore baseline comparability (frequencies, t-test, Chi-square). Primary baseline variables between the response and the non-response group will be performed in order to investigate selective attrition. The primary analysis will be performed according to the intention-to-treat principle. In addition, per-protocol analyses that include only adherent patients of the intervention group and the entire control group will be performed. For all analyses, a two-tailed significance level of p < 0.05 is considered to be statistically significant. All analyses will be carried out with the statistical package STATA.

#### Effectiveness

To determine the short (baseline-3 months) and long term (baseline −12 months) effectiveness of e-Exercise on primary and secondary outcomes, multi-level modelling of repeated measures will be performed controlling for baseline values and relevant confounders such as age, OA location and gender. With multilevel modelling of repeated measures it is possible to correct on one side for dependency of observations within subjects and, on the other side, to take into account the variation between physical therapists [50, 51]. The three-level hierarchy will exist of repeated measurements (level 1), nested within patients (level 2), nested within physical therapists (level 3).

#### Economic evaluation

A cost-utility analysis (CUA) and a cost-effectiveness analysis (CEA) will be performed from the societal and the healthcare perspective. From the societal perspective all costs will be taken into account irrespective of who pays or benefits, whereas solely those borne by the healthcare sector will be included when the healthcare perspective is applied [40]. For the CUA and CEA, missing cost and effect data will be imputed using multiple imputation [52]. The results of the imputed datasets will be pooled using Rubin’s rules [52]. In order to account for the highly skewed nature of cost data, bias-corrected and accelerated bootstrapping with 5000 replications will be used to estimate 95% confidence intervals around the mean differences in costs between the study groups. Incremental Cost-Effectiveness Ratios (ICERs) will subsequently be calculated by dividing the differences in costs between study groups by their respective differences in QALYs for the CUA. For the CEA, ICERs will be calculated by dividing the difference in costs by the difference in PA and physical functioning. The uncertainty surrounding the ICERs will be graphically illustrated by plotting bootstrapped incremental cost-effect pairs on cost-effectiveness planes [53]. Moreover, cost-effectiveness acceptability curves (CEACs) will be constructed to provide a summary measure of the joint uncertainty of costs and effects. CEACs indicate the probability of the e-Exercise intervention being cost-effective in comparison to usual care at different willingness-to-pay values [54]. To test the robustness of the study results, several sensitivity analyses will be performed.

### Timeline

Recruitment of physical therapy practices begun in May 2014. The trial will start in September 2014. Until December 2014 patients are able to enrol the program. The follow-up will last until December 2015. Analysis of the data will start in January 2016.

## Discussion

Scarce health resources and a growing number of patients with OA of the hip and/or knee require cost-effective treatment strategies in patients with OA. The presented RCT will study the (cost)-effectiveness of e-Exercise, an intervention in which face-to-face exercise therapy sessions are partly replaced by a web-based PA intervention. This study is, as far as we know, the first RCT that investigates the (cost)-effectiveness of a blended intervention in patients with knee and hip OA. Therefore, this RCT will provide internationally relevant results regarding the short- and long-term (cost)-effectiveness of an exercise therapy intervention that incorporates modern technologies.

The primary goal of e-Exercise is to improve levels of PA and physical functioning in a cost-effective manner. In addition to our outcome measurements, e-Exercise might have several other benefits beyond the primary scope of this study. First, a number of studies showed that exercise therapy may help to postpone joint replacement surgery [55–57]. For example, in the study of Pisters et al. [56], a 60 month follow-up showed that 20% of the patients from the exercise therapy group underwent total hip surgery, compared to 45% of the patients from the usual care group. The exercise therapy consisted of a 12-week Behavioral Graded Activity treatment [27], which is also incorporated in e-Exercise. Second, it is known that most people with OA of the hip and/or knee suffer from at least one comorbidity, such as cardiovascular diseases and diabetes mellitus. It is presumable that improving PA contribute to patients’ general health status, since PA has several health advantages for these comorbidities [58].

Although the study is well-considered, we take into account potential operational issues. First challenge is the recruitment of sufficient numbers of physical therapists. Since e-Exercise is characterized by fewer physical therapy sessions, physical therapists will receive less reimbursement from health insurances compared to usual care. To deal with this challenge, accreditation points for participating physical therapists will be supplied in order to make study participation more attractive. Another incentive is that physical therapists keep their access to the website after the study is finished. The second challenge is the non-usage of the web-based part of e-Exercise. Previous studies have indicated that participants in online interventions are less motivated and feel less pressure to continue compared to traditional face-to-face interventions [59]. However, in order to stimulate website usage, we will incorporate email reminders into the program. But most importantly, since this study concerns a blended intervention, which is a combination of face-to-face contact with e-health, e-Exercise is expected to maximize adherence compared to self-guided internet interventions [17, 23].

There are several strengths in the design of this study. First, we elaborate on the study results of Joint2Move [24]. This intervention showed to be an effective web-based intervention for patients with OA of the hip and/or knee and will be the fundament for e-Exercise. Second, the primary outcome measurements PA and physical functioning will be measured subjectively (questionnaires) and objectively by means of accelerometers and the timed “Up & Go test”. Third, the 12-month follow-up will result in data about long-term effectiveness. The last strength is that e-Exercise will be evaluated in daily physical therapy practice, the setting in which the intervention will be implemented after the presented trial. Therefore, user experiences can be used in order to improve e-Exercise and to facilitate implementation.
